# Inositol phosphates dynamically enhance stability, solubility, and catalytic activity of mTOR

**DOI:** 10.1016/j.jbc.2024.108095

**Published:** 2024-12-18

**Authors:** Lucia E. Rameh, John D. York, Raymond D. Blind

**Affiliations:** 1 Department of Biochemistry and Molecular Biology, University of South Alabama, Mobile, Alabama, USA; 2 Department of Biochemistry, Vanderbilt University School of Medicine, Nashville, Tennessee, USA; 3 Department of Medicine, Division of Diabetes, Endocrinology and Metabolism, Vanderbilt University Medical Center, Nashville, Tennessee, USA

**Keywords:** inositol phosphates, mTOR, mTOR complex, inositol hexakisphosphate, kinase, enzyme kinetics, kinetics, signaling

## Abstract

Mechanistic target of rapamycin (mTOR) binds the small metabolite inositol hexakisphosphate (IP_6_) as shown in structures of mTOR; however, it remains unclear if IP_6_, or any other inositol phosphate species, function as an integral structural element(s) or catalytic regulator(s) of mTOR. Here, we show that multiple, exogenously added inositol phosphate species can enhance the ability of mTOR and mechanistic target of rapmycin complex 1 (mTORC1) to phosphorylate itself and peptide substrates in *in vitro* kinase reactions, with the higher order phosphorylated species being more potent (IP_6_ = IP_5_ > IP_4_ >> IP_3_). IP_6_ increased the V_MAX_ and decreased the apparent K_M_ of mTOR for ATP. Although IP_6_ did not affect the apparent K_M_ of mTORC1 for ATP, monitoring kinase activity over longer reaction times showed increased product formation, suggesting inositol phosphates stabilize the active form of mTORC1 *in vitro*. The effects of IP_6_ on mTOR were reversible, suggesting IP_6_ bound to mTOR can be exchanged dynamically with the free solvent. Interestingly, we also observed that IP_6_ could alter mTOR electrophoretic mobility under denaturing conditions and its solubility in the presence of manganese. Together, these data suggest for the first time that multiple inositol phosphate species (IP_6_, IP_5_, IP_4_, and to a lesser extent IP_3_) can dynamically regulate mTOR and mTORC1 by promoting a stable, more soluble active state of the kinase. Our data suggest that studies of the dynamics of inositol phosphate regulation of mTOR in cells are well justified.

Inositol hexakisphosphate (IP_6_), also known as phytic acid or phytate, is a ubiquitous small metabolite found in many organisms, from yeast to mammals (1). IP_6_ and other inositol phosphate molecules comprise a family of signaling molecules derived from the cyclic polyhydroxy alcohol myo-inositol, which can be phosphorylated at six different positions of the inositol ring, generating six different species and several isomers of each for a total of 64 possible members, with 30 different species detected in cells (2). IP_6_, the fully phosphorylated form of inositol phosphate is also the most abundant, with cellular concentrations in the range of 24 to 47 μM, as recently confirmed by capillary electrophoresis coupled to electrospray ionization mass spectrometry analysis (3), and reaching 1 mM in plant seeds, which is equivalent to 1% of its dry weight (2). IP_6_ can be further phosphorylated to generate the pyrophosphorylated forms IP_7_ and IP_8_ (4).

The role of IP_6_ in protein function is complex, with reports showing that this small molecule can serve as a competitive or allosteric regulator of enzyme activity, such as described for casein kinase two (5), Bruton’ tyrosine kinase (6), *Yersinia* outer-protein J (7), and histone deacetylases (8). IP_6_ serves as a mediator of protein–protein binding to facilitate intramolecule or intermolecule complexes such as for mixed lineage kinase domain like (9) and Cullin/COP9 (10) and protein oligomerization as for fibrinogen (11). Furthermore, IP_6_ can function as a structural cofactor to promote proper protein folding. In fact, many X-ray crystallographic structures have shown unexpected electron densities consistent with IP_6_, present in the core of the protein (12). Some of them have been confirmed to be IP_6_ by mass spectrometry. As a structural cofactor, IP_6_ association with its protein target must be tight and long-lived and must be buried within the core of the protein, as described for ADAR2 (13).

Although less abundant than IP_6_, other inositol phosphate species have been shown to play critical roles in cell signaling as well. For example, IP_3_ (inositol-1,4,5-P_3_) is well known for its role as a second messenger for various growth factor signals that regulate intracellular calcium release, and IP_4_ (inositol-1,4,5,6-P_4_) was recently shown to allosterically regulate histone deacetylases, through direct binding (14). Some of the roles of inositol phosphates in protein regulation are specific to a certain phosphorylated species and/or isomer while others are shared between multiple species.

Cryo-EM studies and retrospective analysis of crystallographic structures revealed that IP_6_ cocrystalizes with mechanistic target of rapamycin (mTOR) (15, 16), a serine/threonine kinase which is the core catalytic subunit of two complexes, mechanistic target of rapmycin complex 1 (mTORC1) and mechanistic target of rapamycin complex 2 (mTORC2). While both complexes require the accessory protein mLST8 (mammalian homolog of protein Lethal with Sec 13), mTORC1 is characterized by the regulatory subunit Raptor and mTORC2 by Rictor. mTORC1 is a nutrient-sensing kinase that signals for increase in anabolic processes in times of nutrient abundance (17, 18). The combination of growth factors and nutrients, such as amino acids, lipids, and glucose, activates mTORC1 at the lysosomal surface by promoting translocation of mTORC1 from the cytosol to the lysosomal surface and activation of lysosomal RHEB (RHEB/GTP), an allosteric activator of mTORC1 (19). However, RHEB-independent mechanisms for mTORC1 activation must exist, especially considering that mTOR and its substrates are found in different organelles and subcellular locations. In fact, phosphatidic acid was recently shown to activate mTOR in the absence of RHEB (20). To this date, there are no soluble small metabolite shown to directly regulate mTOR catalytic activity.

Interestingly, IP_6_ was found within a highly positive pocket formed by the FAT domain of mTOR referred to as the I-site (16). The FAT domain of mTOR forms a C-shaped solenoid structure that surrounds the N- and C-lobes of the kinase domain and was shown to participate in RHEB/GTP induced conformational changes that culminate in kinase activation (21). Mutation of two or three of the residues that coordinate IP_6_ binding inside the FAT domain was used to address the role of IP_6_ in mTOR complex formation and activity. In one study, mutation of two lysines within the I-site (K1753/1788E) abolished *in vitro* kinase activity of truncated mTOR (15), whereas in another study in which mTOR activity was measured in the presence of its regulatory subunits (LST8, Rictor and SIN1), the same mutations had no impact on complex formation or kinase activity (16). These seemingly contradictory results led Scaiola *et al*. to conclude that IP_6_ binding to mTOR is dispensable for kinase function when the regulatory subunits are present. Both groups proposed that IP_6_ plays a structural role by allowing proper folding of the kinase domain. Surprisingly, neither study addressed whether exogenous IP_6_ can enhance mTOR kinase activity and whether this role of IP_6_ can be fulfilled by other inositol phosphate species.

Here, we examined the impact of various inositol phosphate species on mTOR kinase activity and stability/solubility, in the context of mTOR alone or complexed with its regulatory subunits LST8 and Raptor (mTORC1). The data suggest that exogenous IP_6_, IP_5_, IP_4_, and to a lesser extent IP_3_ enhanced mTOR and mTOR/LST8/Raptor autophosphorylation or phosphorylation of exogenous peptide substrates in a concentration-dependent and saturable manner. This could partially be explained by an increase in solubility/stability of the enzyme. Enzyme kinetics showed that IP_6_ increases V_MAX_ and decreases the K_M_ for ATP of mTOR alone but not of mTOR complexed with LST8 and Raptor. Instead, IP_6_ enhanced mTOR/LST8/Raptor rate of catalysis during prolonged incubations, consistent with enhanced stability. The effect of IP_6_ on mTOR was reversible, indicating a dynamic interaction between the enzyme and this small metabolite, consistent with a regulatory rather than structural role for IP_6_.

## Results

### Higher order inositol phosphates increase autophosphorylation and peptide phosphorylation by mTOR and mTOR/LST8/Raptor

**Figure 1:**
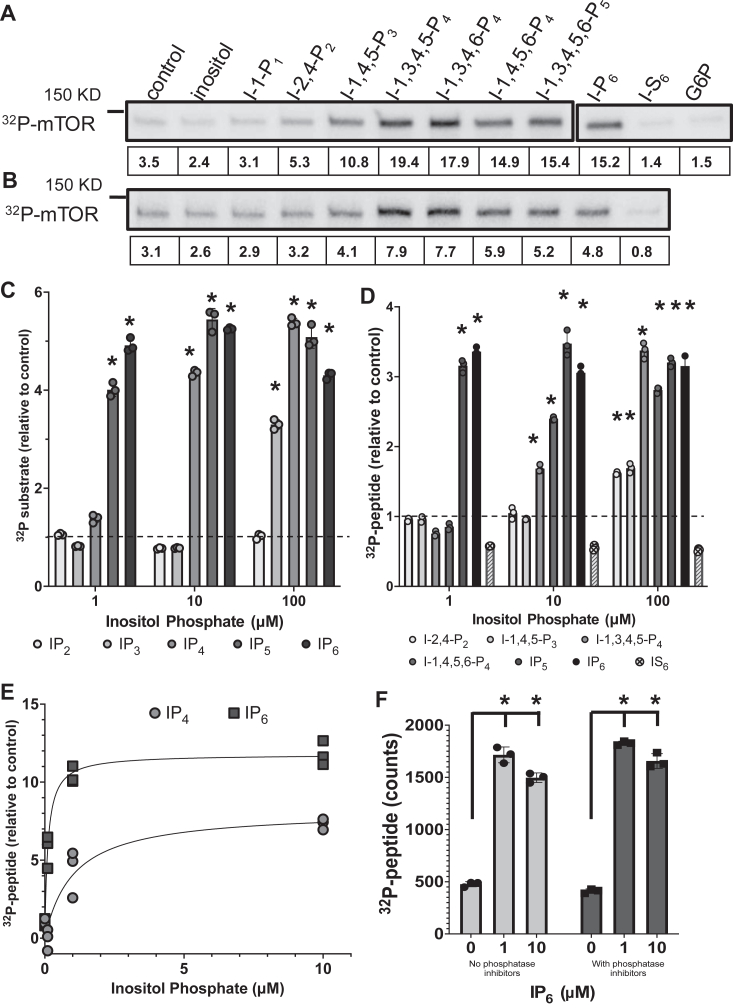
**Inositol phosphates increase auto and peptide phosphorylation by mTOR in a concentration-dependent manner.***A*–*F*, mTOR (*A*, *C*, *E*, and *F*) or mTOR/LST8/Raptor (*B* and *D*) were incubated with [^32^P] γ-ATP in kinase reaction. (*A* and *B*) Autophosphorylation of mTOR and mTOR/LST8/Raptor with or without 100 μM of inositol or inositol phosphate species, inositol hexakis-sulfate (IS_6_), or glucose-6-phosphate (G6P), as shown by phosphoimager images of SDS-PAGE and quantification of the radiolabeled mTOR bands (shown below each band). Coomassie staining of the gels are shown on Fig. S1, *A* and *B*. *C*–*F*, peptide kinase assays in the presence of various concentrations of inositol phosphate species, as indicated. In *C* and *D*, data were normalized against the control (without inositol phosphates, *dotted lines*). Phosphorimager images of the spotted W3 papers for *C* and *D* are shown in Fig. S2, *A* and *B*. In *F*, peptide kinase reactions were done in the presence or absence of the phosphatase inhibitors, NaFl, ß-glycerophosphate, and nor-cantharidin. Data shown are the scattered plot with mean and standard deviations of quantified triplicate spots. (∗) indicate that the increase above control was statistically significant (*p* < 0.0001), using student’s *t* test (two-tails, unpaired). The *p* values for ANOVA analysis of each concentration group were (C) *p* = 2.677^−13^ (1 μM group); *p* = 7.1224^−14^ (10 μM group); *p* = 6.9552^−12^ (100 μM group) and for (*D*), *p* values were *p* = 8.233^−17^ (1 μM group); *p* = 1.0125^−13^ (10 μM group); and *p* = 1.3263^−14^ (100 μM group). mTOR, mechanistic target of rapamycin; NaFl, sodium fluoride.

To better understand how IP_6_ and other inositol phosphate species alter mTOR catalytic activity, we performed autokinase assays using recombinant N-terminal truncated mTOR by itself or co-expressed with LST8 and Raptor (see materials and methods) and radiolabeled-ATP in the presence or absence of exogenous inositol or inositol phosphate species during 1 h incubation time. Radiolabeled phosphate incorporation into both mTOR and mTOR/LST8/Raptor were enhanced several-fold by 100 μM exogenous IP_4_, IP_5_, or IP_6_, but not by inositol, IP_1_, or IP_2_ (Fig. 1, *A* and *B*), despite equal amount of protein being added to all these reactions (Fig. S1, *A* and *B*). The inositol phosphate–dependent increase in autophosphorylation was more robust in mTOR alone (about 6-fold) than in mTOR co-expressed with LST8 and Raptor (about two to 3-fold). IP_3_ also enhanced mTOR and mTOR/LST8/Raptor autophosphorylation although to a lesser extent than the higher phosphorylated forms of inositol. Three different isoforms of IP_4_ were tested with similar outcomes (Fig. 1, *A* and *B*). As a control, we used glucose-6-phosphate and inositol hexa-kis-sulphate (IS_6_) which like IP_6_ is highly negatively charged. However, IS_6_ was unable to enhance phosphate-labeled mTOR (Fig. 1, *A* and *B*), showing that the effect of IP_6_ on mTOR was not mimicked by another highly negatively charged small molecule. These results were confirmed using lower, more physiological concentrations of inositol phosphates (10 μM) in reactions containing higher concentration of mTOR for normalization (Fig. S1, *C* and *D*). These data suggest that multiple inositol phosphate species enhance phosphorylation of mTOR and mTORC1 in autokinase reactions.

To more thoroughly characterize the effect of inositol phosphates on mTOR, we measured radiolabeled phosphate incorporation into peptide substrates in the presence of various concentrations of inositol phosphates, again using radiolabeled ATP during 1 to 2 h assays (Fig. S2, *A* and *B*). We used a peptide substrate derived from the phosphorylation site in 4EBP (eukaryotic translation initiation factor 4-E binding protein), a *bona fide* mTORC1 substrate, which has been successfully used in other studies of mTOR catalytic activity (22). IP_5_ and IP_6_ at 10 μM maximally increased peptide phosphorylation by mTOR or by mTOR/LST8/Raptor as compared to vehicle control (Fig. 1, *C* and *D*). At 0.1 μM, IP_6_ effect was submaximal, showing a concentration-dependent effect (Figs. 1*E* and S2*C*). Using nonlinear curve fit, we calculated an EC50 between 0.07 and 0.17 μM for the IP_6_ effect on mTOR in this peptide phosphorylation assay. IP_4_ (I-1,4,5,6-P_4_) enhanced substrate phosphorylation to a similar extent as IP_5_ and IP_6_, but higher concentrations of IP_4_ were required (EC50 between 0.44 and 2.4 μM) and at least 10 μM for maximal IP_4_ effect (Figs. 1, *C*–*E*). Interestingly, IP_4_ at 10 μM was as efficient at activating mTOR autokinase as IP_5_ or IP_6_ (Figs. 1, *A* and *B* and S1*C*), indicating that autokinase and kinase toward exogenous substrate may be distinctly regulated by these inositol phosphate species. The effect of IP_3_ on substrate phosphorylation by mTOR and mTOR/LST8/Raptor was submaximal even at 100 μM (Fig. 1, *C* and *D*). These data suggest that multiple inositol phosphate species enhance mTOR and mTORC1 peptide phosphorylation in equilibrium reactions.

Since IP_6_ had been previously shown to inhibit serine-threonine phosphatases type 1, 2A, and 3 (23) with K_M_s around 10 μM, we tested whether the increase in peptide phosphorylation in our assays could be due to IP_6_-dependent inhibition of a phosphatase activity (which could potentially copurify with mTOR). If this was true, we expected to see blunting of the IP_6_ effect when broad spectrum phosphatase inhibitors were present. Addition of sodium fluoride, ß-glycerophosphate, and nor-cantharidin to the mTOR kinase reaction did not decrease the IP_6_ effect (Fig. 1*F*), suggesting that the observed IP_6_ effect is most likely independent of any copurifying phosphatase activity in the reactions.

Thus, subphysiological concentrations of inositol phosphates enhanced mTOR’s and mTOR/LST8/Raptor’s ability to phosphorylate exogenous or intramolecular substrates during *in vitro* assays. The sensitivity of mTOR to exogenous inositol phosphate species was directly proportional to the number of phosphates in the molecule (IP_6_ = IP_5_ > IP_4_ > IP_3_ > IP_2_) but neither glucose-6-phosphate nor IS_6_ had any detectable effect.

### IP_6_ increases mTOR solubility in the presence of manganese

**Figure 2:**
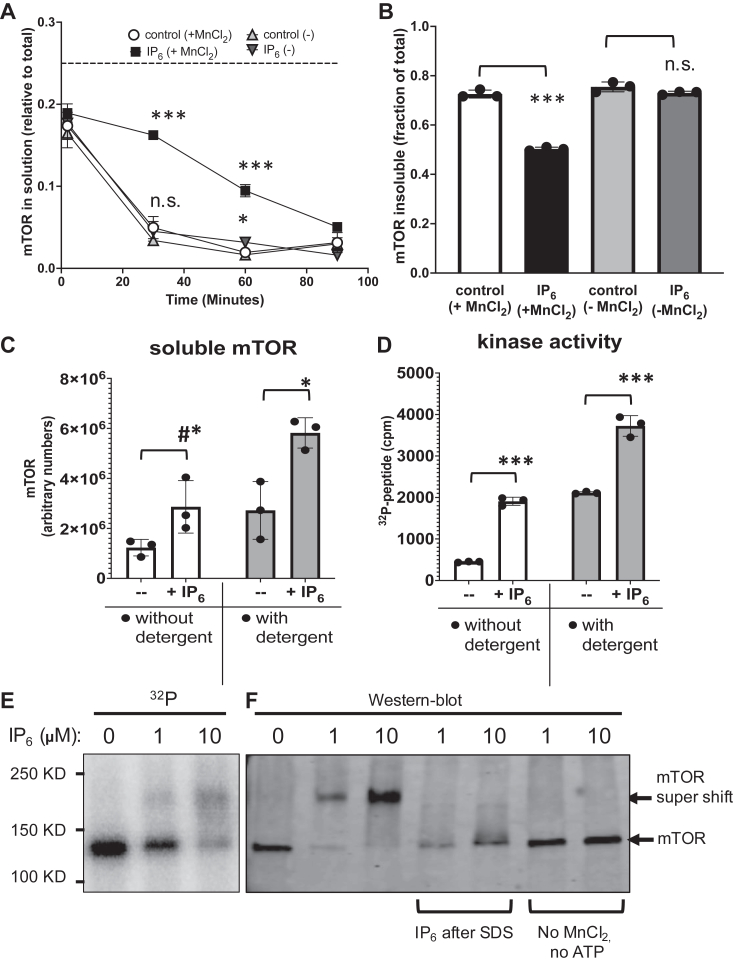
**IP**_**6**_**increases solubility of mTOR and promotes a super-shift in its electrophoretic mobility.***A* and *B*, mTOR kinase reactions with or without IP_6_ (1 μM) and with or without MnCl_2_ (10 mM) were sampled over time and analyzed by western blot for soluble mTOR (*A*), followed by a final extraction of the insoluble material left in the tube after 90 min (*B*). Original western blot images are shown in Fig. S3*A*. In A, the fraction of soluble mTOR (soluble/total) over time of incubation was plotted. Dash line indicates the maximal possible amount of mTOR if completely in solution (0.25) given the volume collected (1/4). In *B*, the fraction of insoluble mTOR (insoluble/total) at the end of the reaction was plotted. In *C* and *D*, mTOR solubility (*C*) and kinase activity (*D*) were assayed with and without 0.1% CHAPS and with or without IP_6_ (10 μM) and samples collected at the end of 90 min for western blot of mTOR (*C*) or [^32^P]-peptide analysis (*D*). Original western blot images are shown in Fig. S3*B*. Shown are the scatter plots with mean and standard deviation of triplicate samples. (∗) indicates that IP_6_ changes are statistically significant as compared to equivalent control without IP_6_ using student’s *t* test (two tails, unpaired), with (A and B) (∗) *p* = 0.017; (∗∗∗) *p* < 0.0002; n.s = nonsignificant; (*C* and *D*) (∗∗∗)*p* < 0.0037; (#∗)*p* = 0.062; and (∗) *p* = 0.015. *E*, phosphorimager image of mTOR after autokinase reaction with [^32^P]-ATP, without (control) or with IP_6_ at the concentration indicated. *F*, western blots of mTOR after autokinase reaction with unlabeled ATP and without (control) or with IP_6_, as indicated. In lanes 4 and 5, IP_6_ was absent at the kinase reaction and added after SDS-loading buffer. In lanes 6 and 7, ATP and MnCl_2_ were not added to the kinase reaction, but only MnCl_2_ was shown to be necessary for the super-shift (see Fig. S4*A*). In *E* and *F*, no EDTA was used to stop the reactions. mTOR, mechanistic target of rapamycin; IP_6,_ inositol hexakisphosphate.

We noticed that in the absence of inositol phosphates, mTOR activity plateaued at around 10 to 15 min (Fig. S2*D*). We did not suspect that mTOR was being proteolytically cleaved because no fragments of mTOR were observed after 1-hour incubations (Fig. S1, *A*–*C*), and our assays regularly contained protease inhibitors. Thus, we suspected that solubility of mTOR could decrease over time. To examine this possibility, we collected one quarter of the supernatant of the kinase reactions at different incubation times. mTOR solubility decreased rapidly over time with 68% (0.17/0.25) of mTOR being soluble at time zero (samples on ice) down to only 16% (0.04/0.25) soluble after 30 min at 30 °C (Figs. 2*A* and S3*A*). When IP_6_ was added to the reaction, 64% (0.16/0.25) of mTOR was still in solution after 30 min, which is a 4-fold increase as compared to control. Concomitant with the increase in IP_6_-dependent mTOR solubility, we observed a decrease in the mTOR fractions that were out of solution (only extractable by SDS/boiling) when IP_6_ was present (Figs. 2*B* and S3*A*). IP_6_ forms a high affinity complex with divalent cations, including manganese (24). Interestingly, when MnCl_2_ was omitted from the reactions, the IP_6_ effect on mTOR solubility was negligible, with only 18% (0.045/0.25) in solution after 30 min at 30 °C (Figs. 2, *A* and *B* and S3*A*). Next, we compared the effect of IP_6_ with CHAPS, a detergent that is commonly used to solubilize mTOR *in vitro*. IP_6_ at 10 μM had a similar effect as 0.1% CHAPS by doubling the fraction of mTOR in solution (Figs. 2*C* and S3*B*). Both, CHAPS and IP_6_ increased mTOR kinase to the same extent (Fig. 2*D*). Interestingly, IP_6_ further enhanced mTOR solubility and activity in the presence of CHAPS, which is present at 100-fold higher molar concentration than IP_6_, consistent with IP_6_ and CHAPS contributing to mTOR solubility in different ways. It is not clear whether the insoluble mTOR is completely inactive or whether it can contribute to kinase activity to some extent. These data suggest that IP_6_ increases mTOR solubility, likely due to changes in protein conformation.

### Association of inositol phosphates with mTOR in the presence of manganese promotes an electrophoretic mobility shift

While examining the effect of IP_6_ on mTOR or mTOR/LST8/Raptor autokinase, we noticed that when exogenous IP_6_ was added to the reaction and EDTA was not used to stop the reaction, mTOR electrophoretic mobility shifted in denaturing SDS-PAGE to an apparent molecular weight band of about 240 K_Da_, which is 100 K_Da_ higher than expected for the recombinant mTOR, which is around 137 K_Da_ (Fig. 2*E*). Using Western blotting, we confirmed that this IP_6_-dependent higher-shifted band cross-reacted with mTOR antibodies (Figs. 2*F* and S4, *A*–*C*). The formation of this super-shifted mTOR band required MnCl_2_ (Figs. 2*F* and S4*A*) and was abolished by EDTA (Fig. S4*C*). Also note that in Figure 1, *A* and *B*, samples were treated with EDTA, thus no shift was observed. Importantly, formation of this super-shift required heating the proteins at 100 °C prior to SDS-PAGE (Fig. S4*B*), suggesting that the IP_6_ modification of mTOR is denaturation-resistant and therefore must involve some molecular cross-linking. However, when IP_6_ was added after SDS denaturation of mTOR, the super-shift did not form, indicating that proper mTOR folding was required (Fig. 2*F*). IP_6_ induced mTOR mobility shift in a concentration-dependent manner, which was observed with IP_6_ concentrations as low as 1 μM (Fig. 2, *E* and *F*), similar to the dose–response for kinase assays, whereas IS_6_ was unable to drive the formation of this high molecular weight band even at higher concentrations (Fig. S4*B*). IP_4_ and IP_5_ were also able to induce the super-shift in a concentration-dependent manner (Fig. S4, *C* and *D*), as for the kinase assays. Thus, the ability of inositol phosphates to promote mTOR super-shift positively correlates with mTOR’s ability to phosphorylate its substrates. ATP was not required for the IP_6_-dependent mTOR electrophoretic mobility shift (Fig. S4*A*), indicating that autophosphorylation cannot explain the shift.

We do not completely understand the nature of the mTOR super-shift, but its ability to survive denaturing conditions highly suggests that it involves the formation of covalent bonds. Since SDS-PAGE mobility is determined by both the molecular mass and the net charges of the protein complexed with SDS, a large decrease in electrophoretic mobility could be attributed to either the formation of a bigger complex (*e*.*g*., due to cross-linking of mTOR dimers) or gross exclusion of SDS molecules (*e*.*g*., due to formation of pockets within mTOR) or both. The mTOR shift we observed with IP_6_ was slightly lower than the expected molecular weight for an mTOR homodimer. Additionally, we noted that IP_4_ induced a lower mobility shift on mTOR than IP_5_ or IP_6_ (Fig. S4, *C* and *D*). Thus, we favor a model in which inositol phosphate binding to mTOR mediate heat-dependent crosslinking of mTOR residues and formation of pockets that exclude SDS molecules, slowing down mTOR’s mobility on SDS-PAGE. We propose that the mTOR super-shift reveals molecular interactions between mTOR and inositol phosphates and suggest that it could be used as a surrogate assay for measuring inositol phosphate binding to mTOR.

### IP_6_ increases mTOR V_MAX_ and decreases K_M_ for ATP

**Figure 3:**
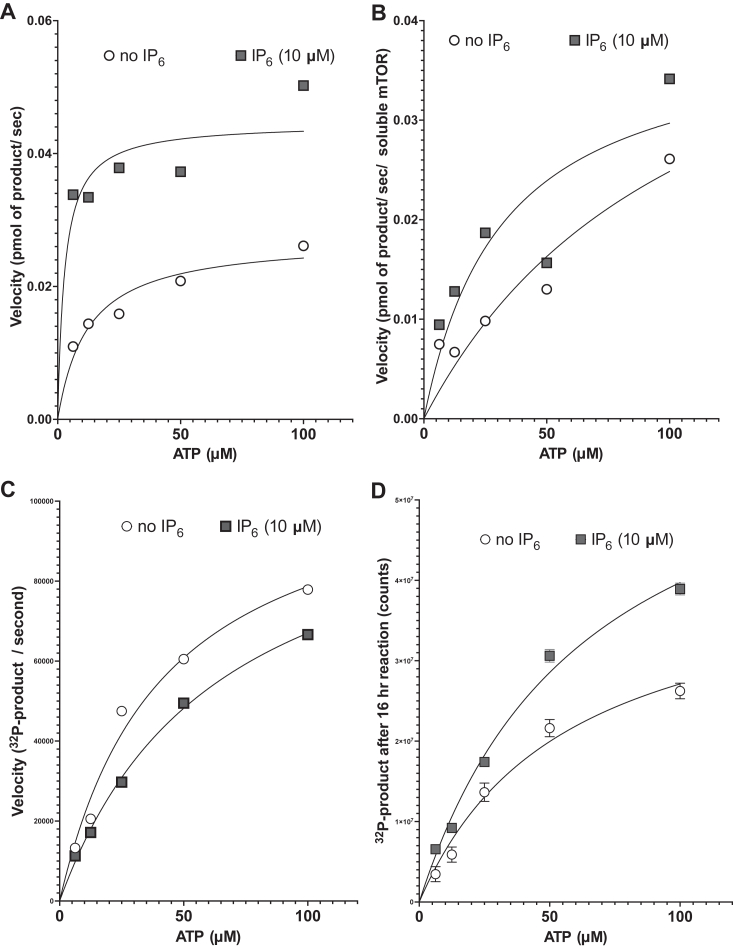
**IP**_**6**_**increases V**_**MAX**_**and decreases the apparent K**_**M**_**of mTOR for ATP, but does not affect mTOR/LST8/Raptor apparent K**_**M**_**for ATP.***A*–*B*, Michaelis–Menten plots of initial velocity of mTOR without (*open circles*) or with 10 μM IP_6_ (*closed squares*) as a function of ATP concentration. In *B*, data were normalized by the measured relative levels of soluble mTOR at the end of the assay. The lines show the nonlinear regression curve fit, and calculated K_M_ and V_MAX_ are shown on Table 1. *C*, Michaelis–Menten plots of initial velocity of mTOR/LST8/Raptor without (*open circles*) or with 10 μM IP_6_ (*closed squares*) as a function of ATP concentration. Nonlinear regression curve fit showed a relative K_M_ for ATP of 41.5 and 64 μM for reactions without or with IP_6_, respectively and V_MAX_ of 1.11 x 10^5^ and 1.09 x10^5^ (counts/sec), respectively. *D*, product formed by mTOR/LST8/Raptor over prolonged incubation (16 h) as a function of ATP concentration. From nonlinear regression curve fit, we extrapolate that mTOR/LST8/Raptor required ATP at 56 μM (without IP_6_) and 64 μM (with IP_6_) for half of the maximal product formation and that the maximal product formed with IP_6_ was 1.54-fold higher than without IP_6_. Velocities were calculated from the plots shown in Fig. S5, *C* and *D*. mTOR, mechanistic target of rapamycin; IP_6,_ inositol hexakisphosphate.

In order to determine how IP_6_ affects mTOR activity toward exogenous peptide, we measured initial enzyme velocity in the presence of various concentrations of ATP (Fig. S5, *A* and *B*). Michaelis-Menten plots (Fig. 3*A*) revealed that IP_6_ at 10 μM decreased the apparent K_M_ for ATP and increased the V_MAX_. The best-fit calculated values for K_M_ for ATP and V_MAX_ with or without IP_6_ are shown on Table 1. Since mTOR solubility is affected by IP_6_, we measured soluble mTOR at the end of the 10-min reaction in each tube and used the relative numbers to normalize the velocity curves and Michaelis–Menten plot (Fig. 3*B*), with the recalculated values shown on Table 1. These values indicate that IP_6_ stabilizes an active conformation of mTOR with higher affinity for ATP and thereby increases the rate of catalysis (V_MAX_).

**Table 1:** The effect of IP_6_ on the K_M_ and V_MAX_ of mTOR

Reactions	Not normalized		Normalized by final soluble mTOR	
	K_M_ (μM)	V_MAX_ (pmol/s)	K_M_ (μM)	V_MAX_ (pmol/s/relative mTOR)
Without IP_6_	12	0.027	110	0.052
With 10 μM IP_6_	2.9	0.045	32	0.039

### IP_6_ prolongs mTOR/LST8/Raptor active state without affecting affinity for ATP

As for mTOR, we measured initial velocities for the complex mTOR/LST8/Raptor with or without exogenous IP_6_ and with varying concentrations of ATP (Fig. S5, *C* and *D*). In contrast to mTOR, the apparent K_M_ for ATP of mTOR/LST8/Raptor complex was not affected by IP_6_ (Fig. 3*C*) and was within the expected range of 50 μM, as previously reported (22). In fact, we calculated that the relative K_M_ for ATP was slightly higher with IP_6_ than without. We also measured velocities with varying concentrations of peptide substrate during 0.5 to 2 h reactions (Fig. S6, *A* and *B*). Michaelis–Menten plots of velocity over substrate concentration showed almost identical curves whether IP_6_ was present or not (Fig. S6*C*). Consistent with a role for LST8 and/or Raptor in stabilizing mTOR kinase, we determined that the complex had higher stability than mTOR alone, with 90 to 100% retention of activity after 2 h preincubation at room temperature (Fig. S7, *A* and *B*). Based on this, we decided to measure whether exogenous IP_6_ can improve catalysis during prolonged incubations, by carrying kinase reactions for several hours (4–16 h). In these long reactions, IP_6_ increased mTOR/LST8/Raptor product formation regardless of ATP concentrations (Fig. 3*D*). Measurements of substrate dependence during extended reactions (8–10 h) also showed an increase in product formation when IP_6_ was present, an effect that was seen regardless of the peptide substrate concentration (Fig. S7*C*). We have calculated that less than 10% of the peptide substrate is phosphorylated even during extended reactions, making it unlikely that substrate availability is limiting. Together, these results suggest that IP_6_ preserves mTOR/LST8/Raptor complex in an active state during extended *in vitro* kinase reactions.

### IP_6_ activation of mTOR is reversible

**Figure 4:**
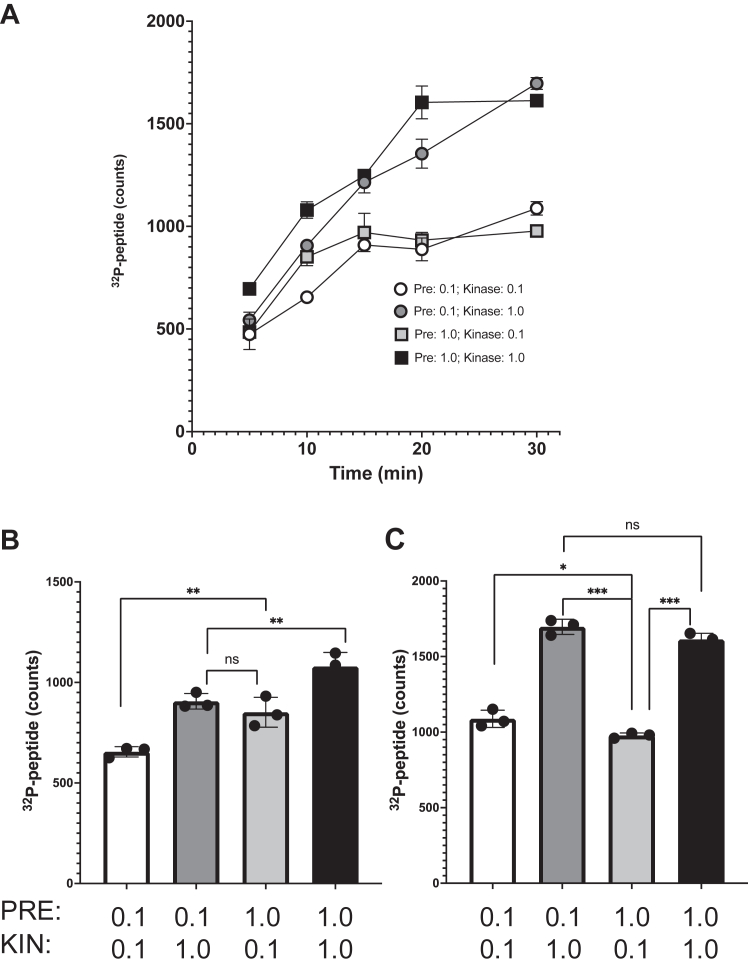
**The IP**_**6**_**effect on mTOR kinase is short-lived.** Peptide phosphorylation over time in kinase assays in which mTOR was preincubated with either low IP_6_ (0.1 μM) or high IP_6_ (1.0 μM) together with MnCl_2_ (10 mM). After preincubation, mTOR was diluted into complete kinase buffer containing either low IP_6_ (0.1 μM) or high IP_6_ (1.0 μM), as indicated. Samples of the reactions were collected at the time point indicated and analyzed for [^32^P]-peptide. Results shown are the mean and standard error of three samples. In *A*, all time points are shown. In *B* and *C*, the scatter plots represent the data from the 10- and 30-min samples, respectively. (∗) indicates statistically significant changes as determined by student’s *t* test (two-tail, unpaired) with (∗) *p* = 0.032; (∗∗) *p* < 0.02; (∗∗∗) *p* < 0.0001. mTOR, mechanistic target of rapamycin; IP_6,_ inositol hexakisphosphate.

IP_6_ is found to be associated with several proteins, and it is thought to function as a structural cofactor to drive proper protein folding. In mTOR, IP_6_ binds to the “I-site”, a pocket within the FAT domain that contains five positively charged residues (16). When these residues were all mutated into negatively charged residues, the protein could not be expressed, supporting the idea that the I-site is necessary for proper mTOR protein folding (15, 16). Intriguingly, our data show that exogenous IP_6_ had a robust effect on the solubility and activity of recombinant mTOR purified from insect cells. If IP_6_ is indeed a structural cofactor for mTOR, we expected that the recombinant mTOR protein used in our assays would copurify with IP_6_, since insect cells have abundant IP_6_. Instead, the data suggested that IP_6_ association with mTOR may be more dynamic than previously appreciated. To test if IP_6_ binding to mTOR is long-lasting, we preincubated mTOR with buffer containing MnCl_2_ and either 0.1 or 1.0 μM IP_6_, which are concentrations that promote submaximal or maximal activation of mTOR, respectively (Fig. 1*E*). Each of these were diluted and assayed for peptide phosphorylation by radiolabeled ATP in reactions that had a final concentration of either 1.0 or 0.1 μM IP_6_. When mTOR was preincubated with IP_6_ at 0.1 μM and kept at 0.1 μM, the kinase reactions were initially slow and plateaued at about 15 min, in contrast to the reactions in which mTOR was assayed with 1 μM IP_6_, which were initially faster and plateau at 20 min (Fig. 4, *A*–*C*). On the other hand, when mTOR was preincubated with 1.0 μM IP_6_ but then diluted to lower 0.1 μM, the kinase reactions were initially faster but then slowed after 10 min and plateaued together with the mTOR that was preincubated and kept at 0.1 μM (Fig. 4, *A*–*C*). These data suggest IP_6_ dissociates from mTOR when diluted out, consistent with mTOR dynamically “sensing” the IP_6_ levels in the kinase reactions.

Together, these data provide the first comprehensive analyses of inositol phosphate regulation of mTOR, suggesting that (i) multiple inositol phosphate species can regulate mTOR and mTOR/LST8/Raptor; (ii) inositol phosphates stabilize an active form of mTOR with higher solubility and affinity for ATP; and (iii) mTOR can dynamically sense IP_6_ concentration, at least in the conditions of *in vitro* kinase reactions tested herein.

## Discussion

mTOR is a large protein with a C-terminal catalytic domain next to the FRB and FAT (Frap, ATP, and TRRAP) domains. The FAT domain of mTOR forms a C-shaped solenoid structure that surrounds the N- and C-lobes of the kinase domain (25). The N-terminal half of mTOR contains HEAT (huntingtin, EF3A, ATM, and TOR) repeats that fold into structures described by the Maier’s et al. as horn and bridge, which participate in mTOR dimerization and binding to Raptor, but not in kinase activity (25). In the presence of LST8 and Raptor, the active-site cleft of mTORC1 is blocked by LST8, Raptor, and the FRB domain of mTOR, which control substrate accessibility and delivery (19, 25). In addition to substrate availability, which is regulated by Raptor, activation of mTORC1 signaling *in vivo* involves amino acid–dependent translocation of mTORC1 to the lysosome, where growth factor–activated RHEB/GTP is present. This small GTPase binds to portions of the N-terminal HEAT and FAT domains and allosterically activates mTORC1 by causing conformational changes in these domains that are transmitted through the FAT domain and ultimately results in alignment of the two lobes and closing of the catalytic cleft (19). As a result, K_CAT_ of the enzyme increases without changes in K_M_ for substrate. These structural findings emphasize the important role of the FAT domain in the regulation of mTOR catalytic activity.

IP_6_ bound to the FAT domain of mTOR was proposed to function as a structural cofactor to aid on mTOR folding when other regulatory subunits are absent (15, 16). Here we show evidence that indicates that mTOR active state is prolonged by IP_4_, IP_5_, and IP_6_ in a dose-dependent and saturable manner, which is more consistent with inositol phosphates having a regulatory role in the stability of active mTOR. Supporting our model, we found that IP_6_/mTOR interaction was reversible and dynamic (Fig. 4). Further, the position of the I-site within the FAT domain is solvent accessible, and in crystallographic studies, the I-site is only partially occupied by IP_6_ (16) suggesting IP_6_ can be exchanged with the environment without complete loss of proper folding. Although it is likely that the effects of inositol phosphate reported here are through the I-site within the FAT domain, this remains to be tested.

Interestingly, our studies revealed that IP_6_ increased the affinity of truncated mTOR for ATP with enhanced rate of catalysis and product formation, which are indicative of allosteric activation and reminiscent of the effect of RHEB on mTOR. We speculate that IP_6_ binding to the FAT improves alignment of the N- and C-lobes, as for RHEB. However, in the presence of LST8 and Raptor, IP_6_ did not increase the affinity of mTOR for ATP, as measured by initial velocity (first 5–10 min). Yet, after several hours of reaction, IP_6_ enhanced product formation by mTOR, whether in the presence of LST8 and Raptor or not. It is possible that IP_6_ copurifies with mTOR/LST8/Raptor but not mTOR alone, which would explain the lack of effect of IP_6_ on initial velocities when mTOR/LST8/Raptor was assayed. However, during prolonged reactions, the effect of exogenous IP_6_ becomes evident, presumably because endogenous IP_6_ in the control samples eventually dissociate from the complex or the complex disassembles. This implies that LST8 and/or Raptor may prevent IP_6_ from dissociating from mTOR. This possibility remains to be tested.

In cells, mTORC1 is regulated by changes in nutrient levels and energy status. We still do not know whether IP_6_ or other inositol phosphates participate in these processes *in vivo*. As a regulator of mTOR in cells, we would expect the levels of cellular IP_6_ to be low and to increase in response to changes in nutrient exposure. In fact, IP_6_ levels in insulin-secreting pancreatic ß-cells were shown to increase in response to glucose stimulation (23). Although cellular levels of IP_6_ are estimated to be around 50 μM (3), far above the levels needed for mTOR activation, as shown here, most of this IP_6_ is likely to be bound to other proteins and therefore unavailable (12). In fact, a recent study using a newly developed IP_6_-specific biosensor revealed that the levels of free-IP_6_ in cells are actually in the low micromolar range (26), which is the concentration range predicted by our studies to be effective at dynamically regulating mTOR. Furthermore, IP_6_ pyrophosphorylation has been implicated in energy sensing in cells (4). Future experiments will address whether inositol pyrophosphates, such as IP_7_ and IP_8,_ can also regulate mTOR kinase.

The Maier’s *et al*. argued that IP_6_ is dispensable for mTORC2 regulation *in vivo* based on the observation that mTORC2 downstream signaling is not affected by knockout or knockdown of the two enzymes that regulate IP_6_, the inositol polyphosphate phosphatase 1 and the IP5 2-kinase (16). However, IP_6_ levels in these cells were not measured. For example, it is possible that inositol polyphosphate phosphatase 1 only regulates a specific pool of IP_6_, given that its subcellular localization is restricted to the inside of the endoplasmic reticulum (27). Another potential explanation for this negative outcome is that IP_5_ or IP_4_ can compensate for the loss of IP_6_ to maintain mTOR active, as indicated by the data presented here. In fact, the *in vivo* concentrations of inositol-1,3,4,5,6-P_5_ (IP_5_) are in the same range as IP_6_ in many cells, although its abundance was found to be less consistent than IP_6_ (3). The IP_4_ isomers, inositol-1,3,4,6-P_4_, inositol-1,3,4,5-P_4_, and inositol-1/3,4,5,6-P_4_ were also detectable through capillary electrophoresis coupled to electrospray ionization mass spectrometry in human cell lines but at lower levels than IP_5_ and IP_6_ (3). Future experiments will address whether mTORC1 and/or mTORC2 is/are affected in cells lacking IP_4_, IP_5_ and IP_6_ and whether fluctuations in the levels of free inositol phosphates can contribute to physiological regulation of these kinase complexes in specific subcellular compartments.

## Experimental procedures

### Compounds

Inositol phosphate and other compounds, inositol-1-monophosphate (IP_1_) (cat# 10007777), inositol-2,4-bisphosphate (IP_2_) (#10008419), inositol-1,4,5-trisphosphate (IP_3_) (#10008205), inositol-1,3,4,5-tetrakisphosphate (IP_4_ designated isomer A) (#60980), inositol-1,3,4,6-tetrakisphosphate (IP_4_ designated isomer B) (#10008442), inositol-1,4,5,6-tetrakisphosphate (IP_4_ designated isomer C or simply IP_4_) (#10007783), inositol-1,3,4,5,6-pentakisphosphate (IP_5_) (#10007784), and IP_6_ (#10008415), were purchase from Cayman Chemical. Please note that three different IP_4_ isoforms were used. When not specified, IP_4_ refers to the inositol-1,4,5,6-tetrakisphosphate isomer. Inositol, IS_6_, and glucose-6-phosphate were from Sigma.

### mTOR proteins

Recombinant SF21/SF9 expressed and purified FLAG-tagged N-terminal truncated mTOR (1362-C-term) and mTORC1 [FLAG-mTOR (1362-C-term), HIS-LST8 and HIS-Raptor] proteins were obtained from Millipore/Sigma by Eurofins DiscoverX products (France), catalog numbers 14-770 and SRP0364, respectively. N-terminal truncated mTOR was previously shown to bind IP_6_ (15) and to behave as full-length mTOR on peptide kinase assays (22). We confirmed the presence of HIS-LST8 and HIS-Raptor in the mTORC1 preparations, with Raptor less abundant than LST8.

### Kinase assays

Unless indicated, all kinase reactions were in Tris pH 7.5 (50 mM), NaCl (100 mM), MnCl_2_ (10 mM), DTT (1 mM), and ATP (10 μM) with or without [^32^P]-ATP (approximately 1 μCi/10 μl). Reactions were typically carried out at 30 °C for 0.5 to 1.5 h, unless indicated. For autokinase reactions, mTOR or mTORC1 were used at 100 ng per reaction (10 ng/μl), and reaction was stopped with 10 to 15 mM EDTA (unless indicated as in Figs. 2, *E* and *F* and S2), before addition of SDS-loading buffer and SDS-polyacrylamide gel electrophoresis (PAGE). Gels were stained with Coomassie stain InstantBlue (Abcam), dried before exposure, and analyzed using a Typhoon (Cytiva) phosphorimager. For the experiment shown on Fig. S1, *C* and *D*, 200 ng of mTOR or mTOR/LST8/Raptor was used per reaction/lane, Coomassie stained bands were visualized using Odyssey (LI-COR) infra-red scanner and analyzed using Image Studio software. Peptide kinase assays were designed based on the work by Sabatini’s *et al*. (22) with modifications and using the following peptides as substrate (HPLC purified, Biomatik): GYDYSTTPGGTGRRRRR (derived from 4EBP S65 phospho-site) and GYFLGFTYVAPGRRRRR (derived from p70S6K T389 phospho-site). Unless otherwise indicated, 4EBP-derived peptide was used at 40 to 100 μM, and mTOR was used at 5 to 10 ng/μl or 33 to 66 nM. For Figures 3, *A* and *B* and 4, CHAPS was used at 0.02% to improve mTOR solubility and further diluted during kinase reactions. Reactions were stopped with EDTA (15 mM) and spotted in triplicates into W3 cation exchange filter paper (from Jon Oakhill, St Vincent’s Institute Medical Research, Australia). Filters were washed in 0.42% H_3_PO_4_, for three 30-min cycles, and left in 0.42% H_3_PO_4_ overnight before air drying. A mixture of buffer and [^32^P]-ATP was spotted as negative control for background detection. The radioactivity present in each spot was quantified using phosphorimager (Typhoon, Cytiva) and Image Studio software or by mixing each spot with scintillation fluid and measuring cpm with scintillation counter (Packard Tricarb, PerkinElmer, and Quantasmart software). For the phosphatase inhibitor experiments (Fig. 1*F*), sodium fluoride (0.5 mg/ml), ß-glycerophosphate (0.5 mg/ml), and nor-cantharidin (5 μM) were used together with protease inhibitor cocktail (Pierce).

### Western blots and solubility assay

Western blots and solubility assays were set up exactly as for the kinase assays using peptide as substrate with the exception that only cold ATP was used. mTOR concentration was 5 ng/μl and was diluted in buffer containing protease inhibitors. All samples were boiled and separated on SDS-PAGE using a precast gradient gel (4–15%, Bio-Rad). Proteins were transferred to nitrocellulose membrane, which was then blocked with 5% milk and blotted using anti-mTOR antibody (Cell Signaling Technologies, cat#2883) as primary antibody. Anti-rabbit conjugated with infra-red dye 680 was used as secondary antibody, and membranes were scanned using the Odyssey infra-red scanner (LI-COR) and analyzed with Image Studio software. For the experiment shown in Figure 2, *A* and *B* (Fig. S3, *A* and *B*), triplicate reactions were set up separately, and 2.5 μl samples were collected for each time point which is equivalent to one-fourth of the total volume. The mTOR leftover in the tubes were extracted with EDTA/SDS loading buffer and considered the insoluble fraction. For the experiment shown in Fig. 2, *C* and *D*, three separate samples of the supernatant (2.5 μl each) were collected and transferred to a new tube containing EDTA (15 mM) and SDS-gel loading buffer. Kinase and solubility reactions were run in parallel.

### Gel shift assays

mTOR electrophoretic mobility shift was assayed as for autokinase assay with [^32^P]-ATP, cold ATP, or no ATP as indicated. Reactions were set up exactly as for kinase reactions, unless indicated otherwise. However, for the super-shift assay, SDS-loading buffer was used to stop the reactions without any EDTA being present. When [^32^P]-ATP was used, mTOR was detected using phosphorimager (Typhoon, Cytiva), and when cold ATP (or no ATP) was used, mTOR was detected using western blot as described above. Typically, less mTOR per lane was used when detection was through western blot (5–10 ng/lane).

## Data availability

All relevant data are contained within the manuscript. Additional information may be requested by contacting the corresponding authors.

## Supporting information

This article contains supporting information.

## Conflict of interests

The authors declare that they have no conflicts of interest with the contents of this article.

## Supporting information

Supporting Figure legends

Sup Fig 1

Sup Fig 2

Sup Fig 3

Sup Fig 4

Sup Fig 5

Sup Fig 6

Sup Fig 7
