# Supplementary material for: Inositol phosphates dynamically enhance stability, solubility, and catalytic activity of mTOR
Source: J Biol Chem. 2024 Dec 18;301(2):108095. doi: 10.1016/j.jbc.2024.108095 (PMC11782818; doi:10.1016/j.jbc.2024.108095)
Supplement: Supporting Figure legends [file mmc1.docx]

**Supporting material**

**Supplemental Figure 1.** Autokinase assays of mTOR and mTOR/LST8/Raptor in the presence or absence of inositol phosphates. (A and B) Coomassie stained images of the gels shown on Figure 1A (A) or 1B (B). Please note that the amounts of mTOR used per lane (100 ng) in these experiments is near the threshold for detection by the Coomassie stain method used and although not all the lanes in panel A show mTOR protein, it demonstrates that there are no excess of mTOR protein in the lanes in which radiolabeled phosphate incorporation was highest. (C) mTOR/LST8/Raptor (left side) or mTOR (right side) autokinase was measured using 200 ng of protein and 10 μM of inositol phosphates, as indicated. Shown are phosphorimager image and infra-red images of the equivalent Coomassie stained gel with quantification and normalization of the counts using Coomassie stained mTOR (D). Also shown are the [^32^P]-LST8 and coomassie stained LST8 bands.

**Supplemental Figure 2.** Peptide kinase assays in the presence of various concentrations of inositol phosphate species, as indicated. mTOR (A, C and D) or mTOR/LST8/Raptor (B) were incubated with [^32^P] γ-ATP and peptide substrate in kinase reaction without (control) or with various concentrations of inositol phosphates, as indicated. (A-B) Phosphorimager images of the spotted W3 papers including control with no inositol phosphates, reactions without peptide or ^32^P-ATP alone. (C) IP_4_ and IP_6_ enhance mTOR phosphorylation of peptide substrate with different affinities. Results shown is the scattered plot with the mean and standard deviations of quantified triplicate spots. (D) mTOR kinase towards 4EBP or p70S6K peptide substrate is short lived. mTOR kinase activity was assay using 4EBP or p70S6K-derived peptide substrate with [^32^P] γ-ATP in kinase reaction without any inositol phosphate. Samples were collected at the time indicated for spotting on W3 paper and analysis of the counts present in the peptides.

**Supplemental Figure 3. IP_6_ increases solubility of mTOR.** (A) Western blot images showing mTOR protein after kinase reactions with unlabeled ATP, with or without IP_6_ (1 μM) and with or without MnCl_2_ (10 mM) that were sampled over time followed by a final extraction of the insoluble material left in the tube after 90 minutes. (B) Western blot images showing mTOR protein after kinase reactions with unlabeled ATP, with or without IP_6_ (1 μM) and with or without 0.1% CHAPS. Samples were collected at the end of 90 minutes for western-blot of using anti-mTOR antibody.

**Supplemental Figure 4.** IP_4_ and IP_6_, but not IS_6_, promote the formation of mTOR super-shift in electrophoretic mobility in a dose-dependent manner. Western-blots (A-C) or phosphorimager image (D) of mTOR after kinase reactions with unlabeled ATP (A-C) or ^32^P-ATP (D), and without (control) or with IP_4_ (C and D), IP_5_ (C), IP_6_ (A-D) or IS_6_ (B), as indicated. In panel A, lane 3, kinase reaction was kept at 0^o^C; lane 4, reaction was carried out without MnCl_2_ or ATP; lane 5 is similar to lane 4 except that MnCl_2_ at 10 mM was present. In panel B lane 6, loading buffer without SDS was used and in lane 7, samples were not boiled at 100^o^C. Unless indicated (panel C), all reactions were stopped with loading buffer and without the addition of EDTA.

**Supplemental Figure 5.** Initial velocities of mTOR (A-B) or mTOR/LST8/Raptor (C-D) in the absence (A and C) or presence (B and D) of IP_6_ (10 μM) and varying concentrations of ^32^P-ATP/ATP, as indicated.

**Supplemental Figure 6.** IP_6_ does not affect mTOR/LST8/Raptor affinity for peptide substrate. (A-B) Velocities of mTOR/LST8/Raptor over 2 hrs incubation in the absence (A) or presence (B) of IP_6_ (10 μM) and varying concentrations of 4EBP peptide substrate. (C) Michaelis-Menten plot of initial velocity (product in counts/second) as a function of peptide substrate concentration. Calculations of relative K_M_ were not possible because V_max_ was not obtained.

**Supplemental Figure 7.** mTOR and mTOR/LST8/Raptor activity after prolonged incubations. mTOR (A) or mTOR/LST8/Raptor (B) were incubated without or with 10 μM IP_6_ at room temperature in kinase buffer containing all components except ATP. At the time indicated, samples were collected, [^32^P]-ATP/ATP was added and assayed for 30 minutes at 30^o^C. Data shown are the mean and standard deviations of triplicate samples spotted on W3 paper and counted in phosphorimager. (C) Product formed by mTOR/LST8/Raptor over prolonged incubation (18 hrs) as a function of peptide substrate concentration, as incdicated.
