# Supplementary figures and images for: Inositol phosphates dynamically enhance stability, solubility, and catalytic activity of mTOR
Source: J Biol Chem. 2024 Dec 18;301(2):108095. doi: 10.1016/j.jbc.2024.108095 (PMC11782818; doi:10.1016/j.jbc.2024.108095)

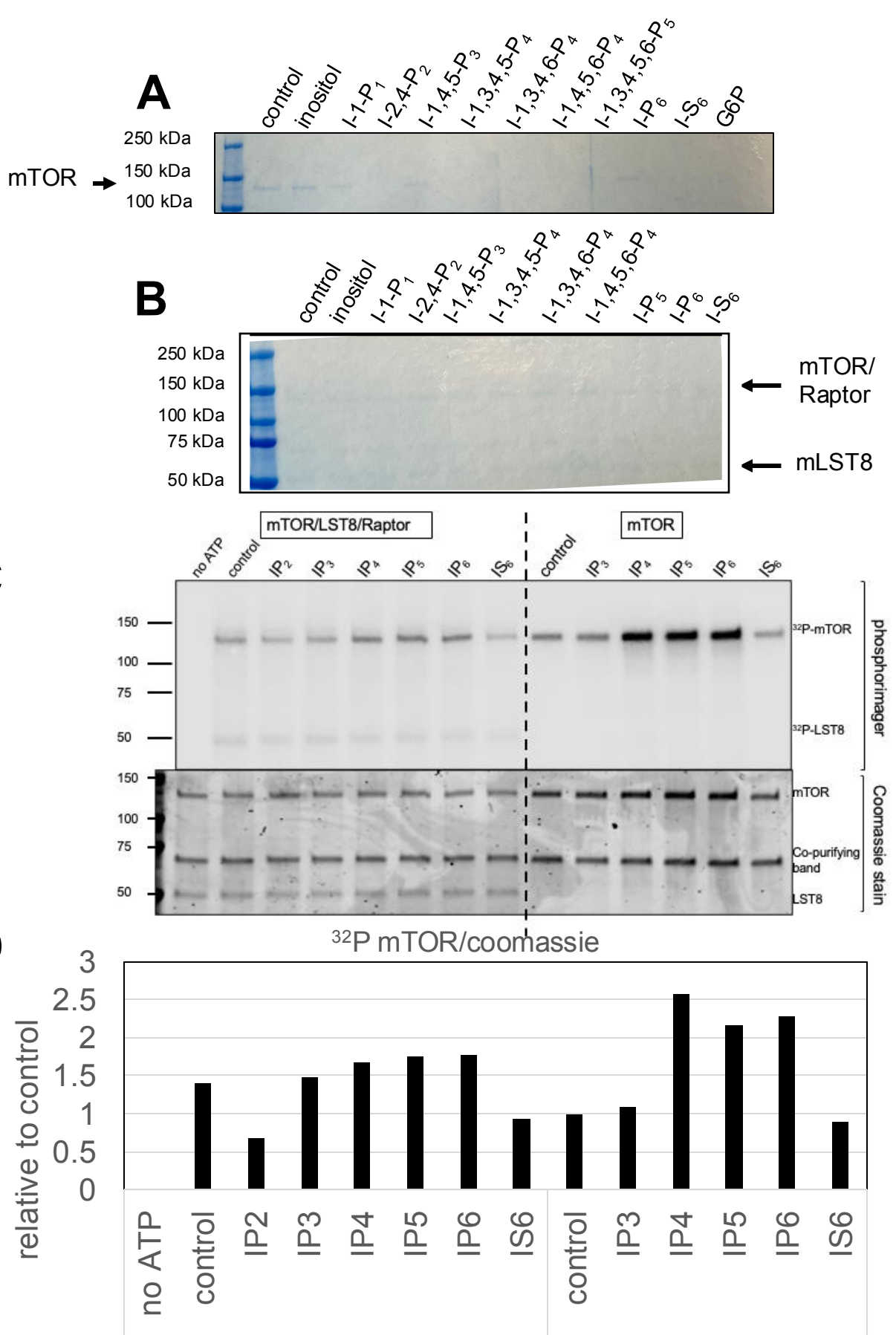

Supplemental Figure 1: Rameh et al

Supplement: Sup Fig 1 [file mmc2.pdf]

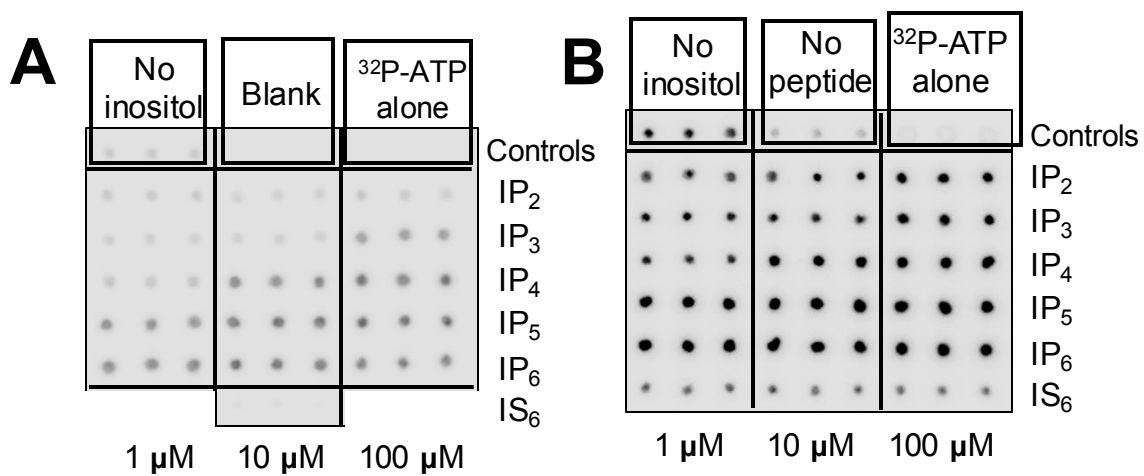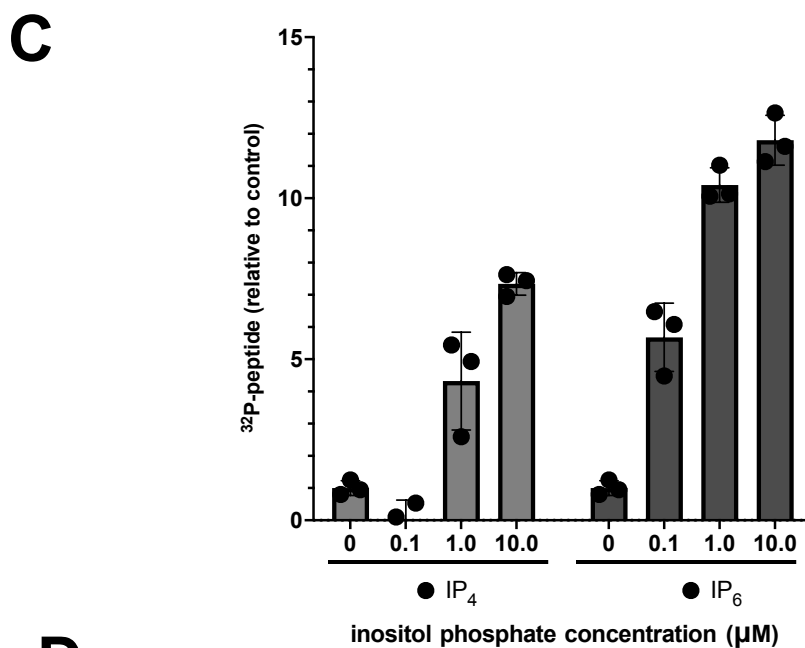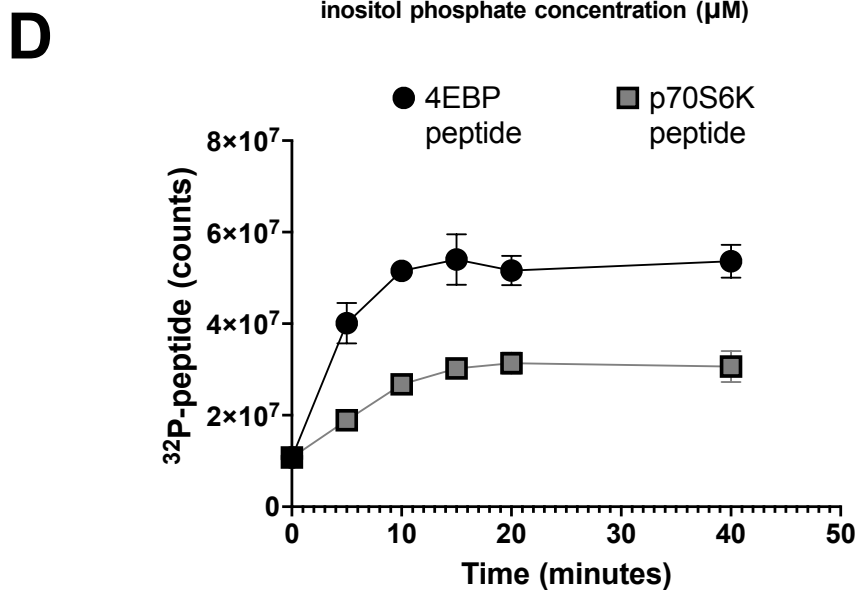

Supplemental Figure 2: Rameh et al

Supplement: Sup Fig 2 [file mmc3.pdf]

**A**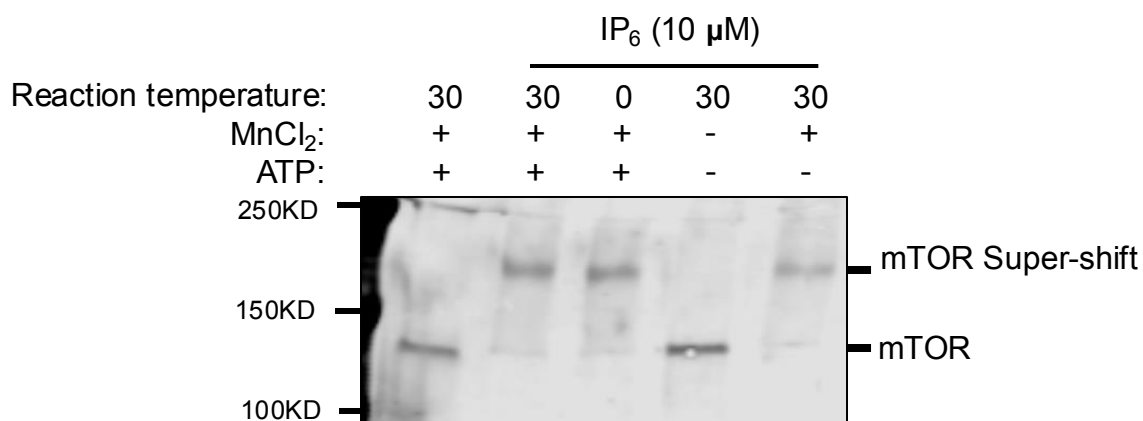**B**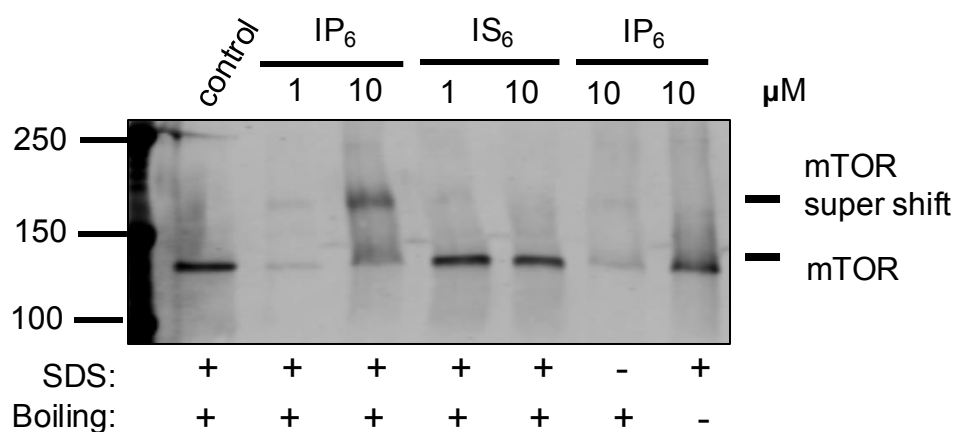**C**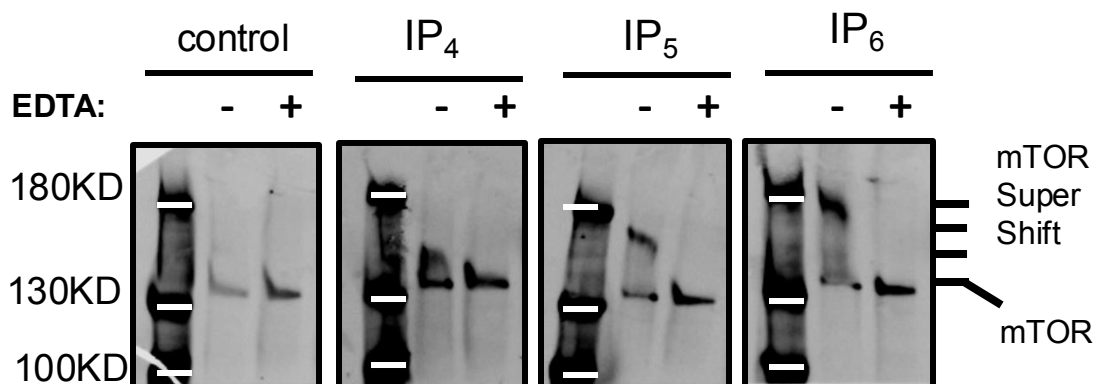**D**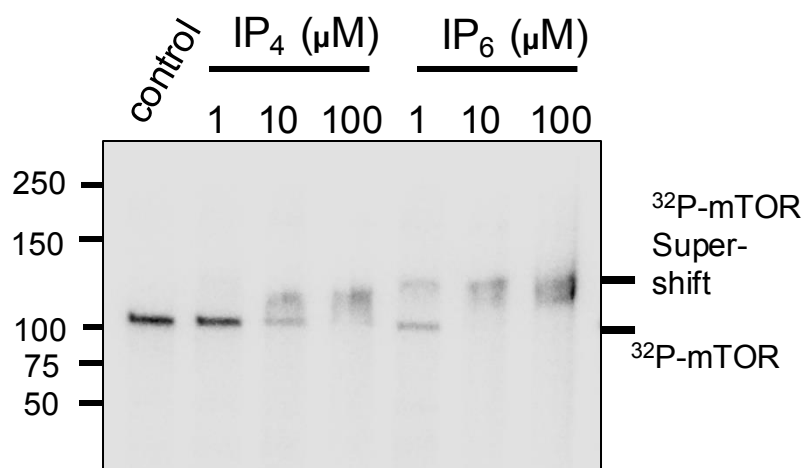

Supplement: Sup Fig 4 [file mmc5.pdf]

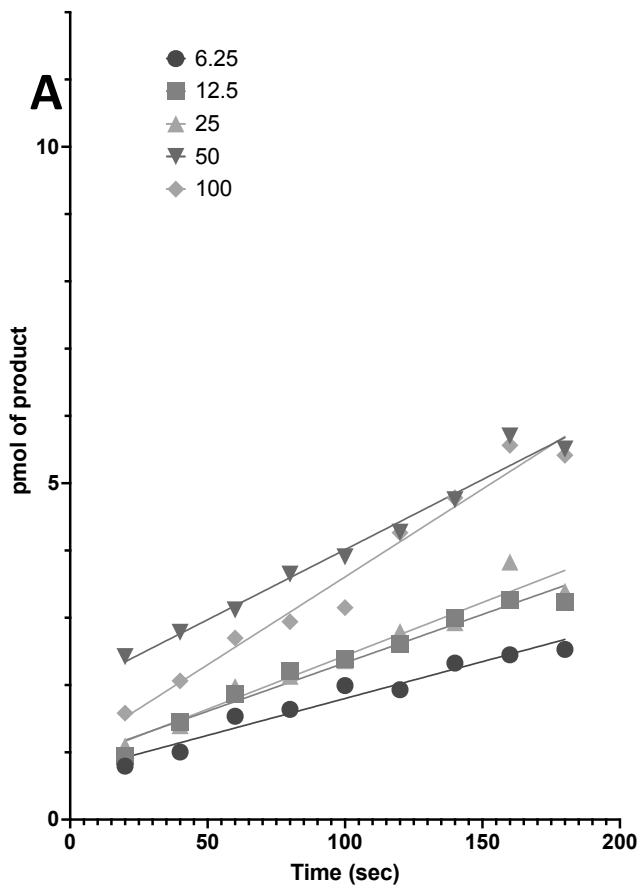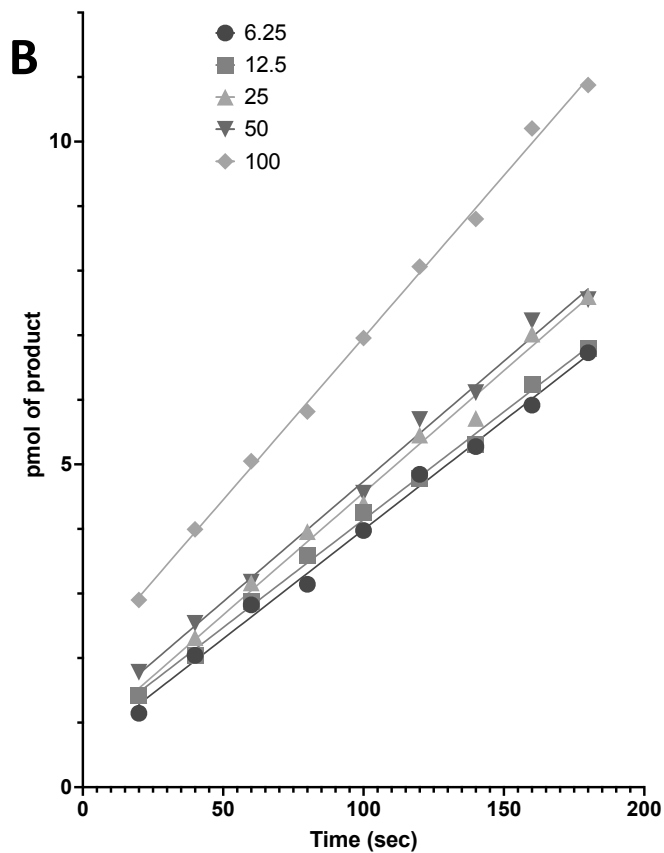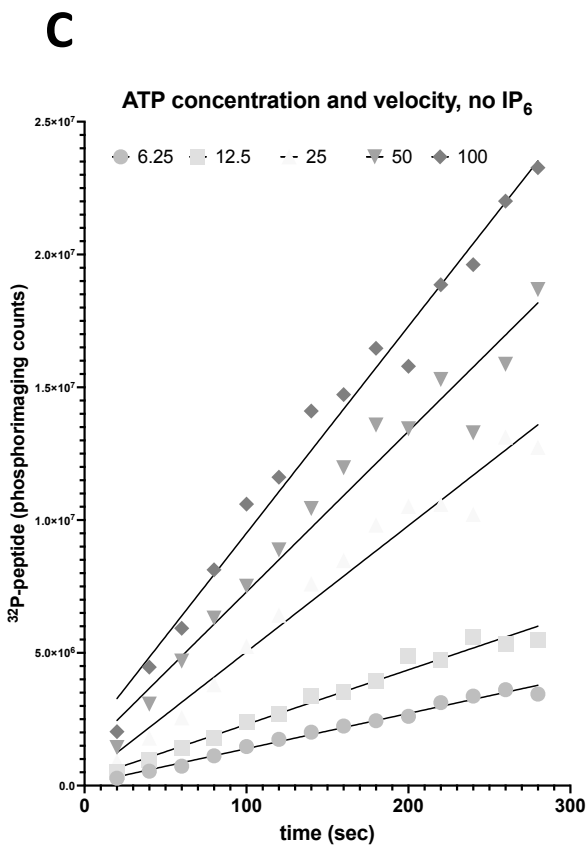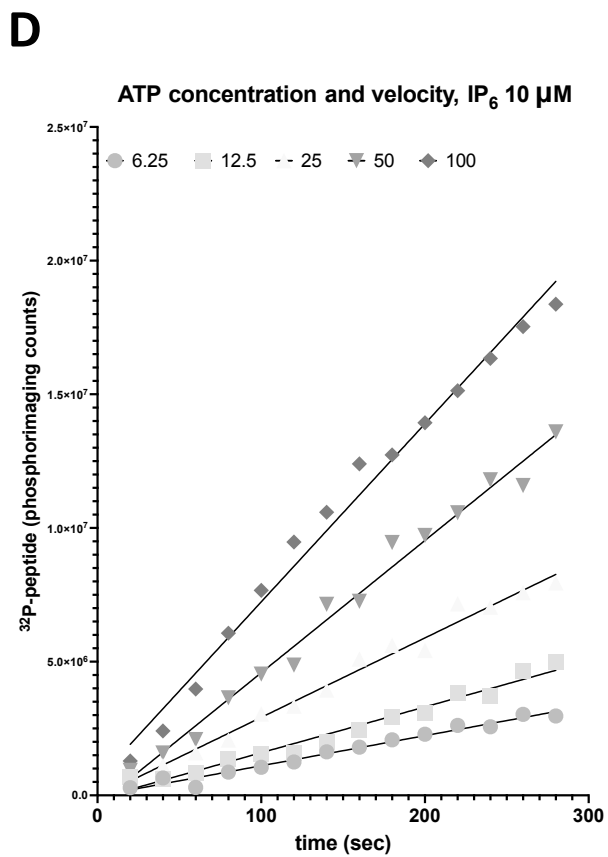

Supplement: Sup Fig 5 [file mmc6.pdf]

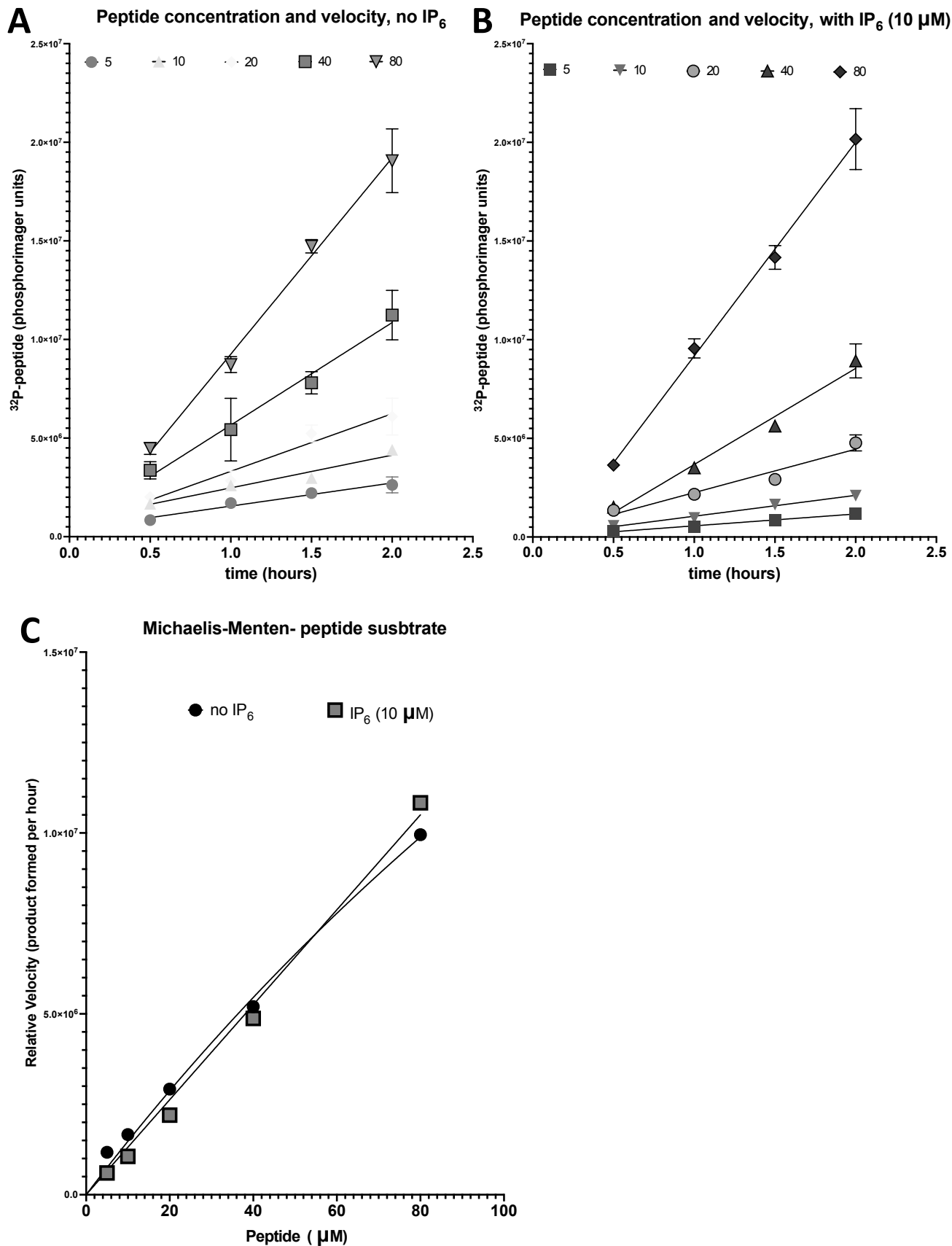

Supplemental Figure 6: Rameh et al

Supplement: Sup Fig 6 [file mmc7.pdf]

**A**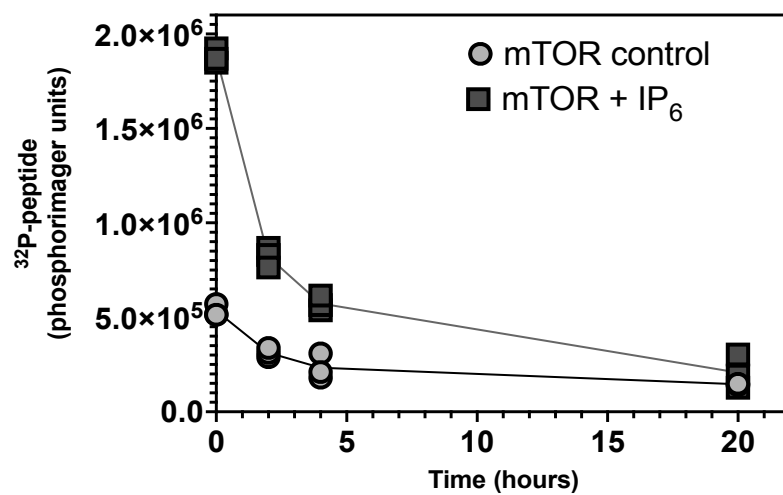**B**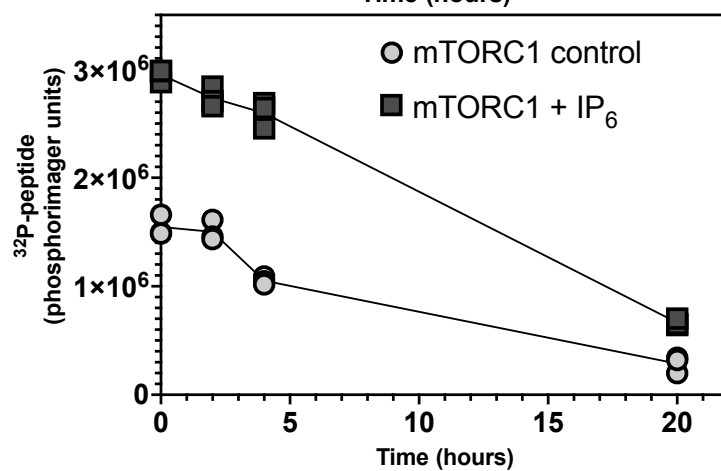**C**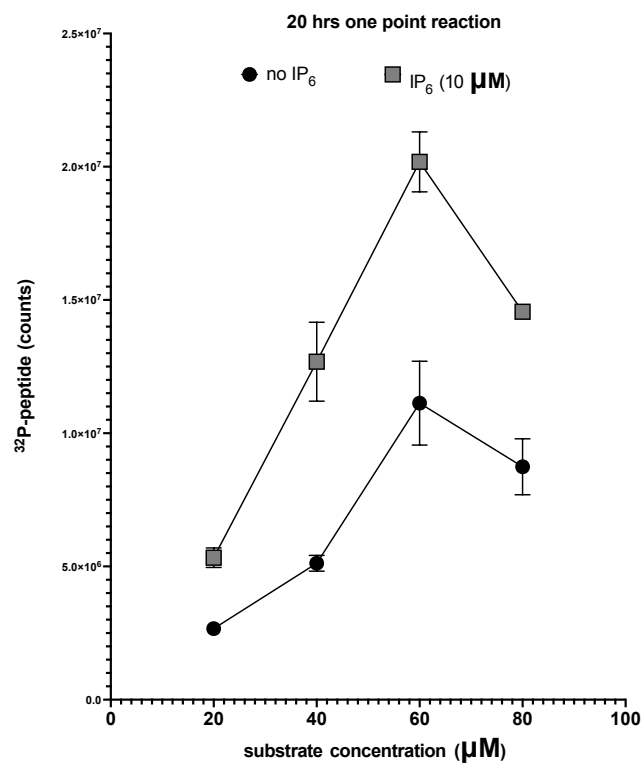

Supplement: Sup Fig 7 [file mmc8.pdf]
