# Supplementary material for: Inositol phosphates dynamically enhance stability, solubility, and catalytic activity of mTOR
Source: J Biol Chem. 2024 Dec 18;301(2):108095. doi: 10.1016/j.jbc.2024.108095 (PMC11782818; doi:10.1016/j.jbc.2024.108095)
Supplement: Sup Fig 3 [file mmc4.pdf]

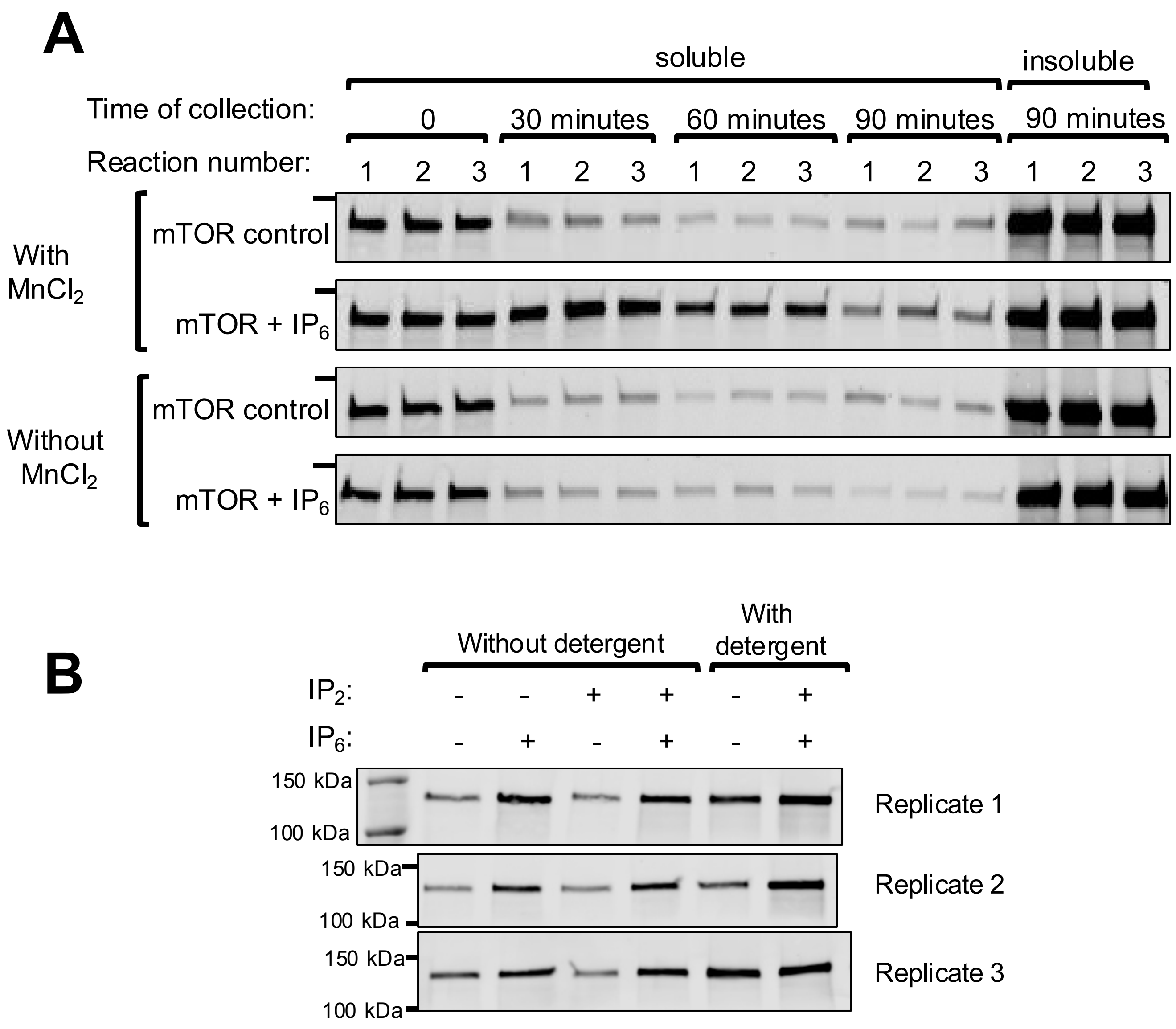

Supplemental Figure 3: Rameh et al
